# Epidemiological characteristics of HIV infected Korean: Korea HIV/AIDS Cohort Study

**DOI:** 10.4178/epih.e2019037

**Published:** 2019-09-03

**Authors:** Yunsu Choi, Bo Youl Choi, Soo Min Kim, Sang Il Kim, June Kim, Jun Young Choi, Shin-Woo Kim, Joon Young Song, Youn Jeong Kim, Dae Won Park, Hyo Youl Kim, Hee-Jung Choi, Mee-Kyung Kee, Young Hyun Shin, Myeongsu Yoo

**Affiliations:** 1 Department of Preventive Medicine, Hanyang University College of Medicine, Seoul, Korea; 2 Institute for Health and Society, Hanyang University, Seoul, Korea; 3 Department of Applied Statistics, Yonsei University College of Business and Economics, Seoul, Korea; 4 Division of Infectious Disease, Department of Internal Medicine, Seoul St. Mary’s Hospital, College of Medicine, The Catholic University of Korea, Seoul, Korea; 5 Department of Internal Medicine and AIDS Research Institute, Yonsei University College of Medicine, Seoul, Korea; 6 Department of Internal Medicine, Kyungpook National University School of Medicine, Daegu, Korea; 7 Division of Infectious Diseases, Department of Internal Medicine, Korea University College of Medicine, Seoul, Korea; 8 Department of Internal Medicine, Yonsei University Wonju College of Medicine, Wonju, Korea; 9 Department of Internal Medicine, Korea University College of Medicine, Seoul, Korea; 10 Department of Internal Medicine, Ewha Womans University School of Medicine, Seoul, Korea; 11 Division of Viral Disease Research Center for Infectious Disease Research, Korea National Institute of Health, Cheongju, Korea

**Keywords:** HIV, Communicable diseases, AIDS-related opportunistic infections, Antiretroviral therapy highly active

## Abstract

**OBJECTIVES:**

To manage evidence-based diseases, it is important to identify the characteristics of patients in each country.

**METHODS:**

The Korea HIV/AIDS Cohort Study seeks to identify the epidemiological characteristics of 1,442 Korean individuals with human immunodeficiency virus (HIV) infection (12% of Korean individuals with HIV infection in 2017) who visited 21 university hospitals nationwide. The descriptive statistics were presented using the Korea HIV/AIDS cohort data (2006-2016).

**RESULTS:**

Men accounted for 93.3% of the total number of respondents, and approximately 55.8% of respondents reported having an acute infection symptom. According to the transmission route, infection caused by sexual contact accounted for 94.4%, of which 60.4% were caused by sexual contact with the same sex or both males and females. Participants repeatedly answered the survey to decrease depression and anxiety scores. Of the total participants, 89.1% received antiretroviral therapy (ART). In the initial ART, 95.3% of patients were treated based on the recommendation. The median CD4 T-cell count at the time of diagnosis was 229.5 and improved to 331 after the initial ART. Of the patients, 16.6% and 9.4% had tuberculosis and syphilis, respectively, and 26.7% had pneumocystis pneumonia. In the medical history, sexually transmitted infectious diseases showed the highest prevalence, followed by endocrine diseases. The main reasons for termination were loss to follow-up (29.9%) and withdrawal of consent (18.7%).

**CONCLUSIONS:**

Early diagnosis and ART should be performed at an appropriate time to prevent the development of new infection.

## INTRODUCTION

The Joint United Nations Programme on Acquired Immune Deficiency Syndrome (UNAIDS) has declared the fight against human immunodeficiency virus (HIV) transmission. It aims to end the HIV epidemic by 2030 and reduce the number of new infections to less than half a million by 2020 [1]. Sustainable development goals have been established and implemented considering the epidemiological characteristics of infected individuals in each country. Moreover, with the help of active development plans, the number of new patients with HIV infection has been steadily decreasing since 1995 [2]. In the 1980s, when HIV/AIDS was first recognized, the mortality rate caused by HIV-related diseases, such as acute infection symptoms, opportunistic infection, and related/defining diseases, was high [3], while with the development of antiretroviral therapy (ART), acquired immune deficiency syndrome (AIDS)-related mortality rate has decreased through therapeutic effects, such as viral suppression and immune level improvement. However, as long-term medication increases the incidence of metabolic diseases such as kidneys, liver, and survival after HIV infection, chronic diseases such as cardiovascular and lung diseases due to aging and death due to HIV-associated cancer increase. In this reason, natural course of HIV/AIDS disease is changing [4-9].

Several countries have been conducting cohort studies to identify the changing nature course of HIV. The first HIV cohort study was the Multicenter AIDS Cohort Study, which began in the USA in 1983 and has been actively recruiting and conducting research on HIV/AIDS guidelines and natural course of HIV/AIDS. The participants of this study are men who sex with men (MSM) and bisexual men, who are at high-risk group of HIV infection. It has the advantage that even possible to participate in HIV seronegative patients, seroconversion mechanism of them can identify in the study [10]. Since then, the number of patients with HIV infection has continuously increased, and various HIV cohort studies have been established in Switzerland [11], the UK [12], and Denmark [13]. However, each cohort has different inclusion criteria, and as pointed out in previous study, race and sex affect the progression of HIV-1 infection [14], country-specific characteristics should be considered in establishing guidelines for care and treatment.

In Korea, the HIV epidemic started with two patients (one Korean and one foreigner) in 1985, and over 100 individuals were known to develop the infection initially in 1995. Since then, the number of HIV new infections has increased steadily, and since 2013, individuals over 1,100 are diagnosed with HIV infection annually [15]. In Korea, to protect of HIV epidemic and control, 90% of HIV-related medical costs are supported by national health insurance, and 10% of deductibles are also supported by the country for those who have registered in Korea Centers for Disease Control and Prevention (KCDC) and receive AIDS registry number [16]. This is to ensure that no patient with HIV infection misses treatment due to financial issues.

It is greatly important to fully understand the characteristics of domestic patients with HIV infection to develop customized medical guidelines and treatment strategies for the patients in each country. The aim of this study to identify and provide evidence of epidemiological features of Korean patients with HIV infection using the only HIV/AIDS cohort data in Korea.

## MATERIALS AND METHODS

### Materials and statistical analysis

Data from the Korea HIV/AIDS cohort study, which included a total of 5,795 observations recorded from 1,442 participants who completed the survey 1 to 5 times (baseline survey and 4 additional visits) from December 2006 to December 2016, were analyzed. To date, 933 individuals are currently participating in the study, excluding 509 participants who dropped out of the study because of death, immigration, or withdrawal of consent, etc. [17].

Descriptive statistics was performed for all data including missing values. For data construction and statistical analysis, SAS Enterprise Guide 7.1 (https://support.sas.com/en/software/enterprise-guide-support.html) was used. The frequencies and percentages of the categorical variables were obtained, and the median and interquartile range (IQR) were calculated for the blood tests that did not show a normal distribution.

### Data collection

Surveys were conducted every 6 months, and the survey period for certain questions that do not show rapid change in a short period was set to 12 months. The baseline survey collected data on events that occurred throughout the lifetime of the patients or after HIV infection diagnosis, and the follow-up surveys collected all data changes since the last survey to establish cohort data. Demographics, such as sex, age, marital status, and transmission route, were determined through self-reporting. A psychological assessment was also conducted through self-reporting: Beck’s Depression Inventory (BDI) consisting of 21 questions in Korean language to assess depression and State-Trait Anxiety Inventory (STAI-X-1) consisting of 20 questions to assess anxiety. The severity of symptoms was determined by dividing the score of the survey result into 4 groups [18-20]. ART and treatment adherence, chronic disease, and other related diseases were evaluated through patient interviews and medical records. The blood test results were considered valid up to 3 months of a particular period of time. Data on AIDS-related/defining diseases that were diagnosed by a specialist from 1 year prior to the date of HIV infection diagnosis to the time of the investigation were collected, and history of opportunistic infections, regardless of HIV infection diagnosis, was analyzed. In the termination survey, interviews of those who completed the study were conducted, and an additional follow-up study was performed, with the help of medical or public records, to investigate death of those who dropped out.

### Ethics statement

This is the same purpose as the Korea HIV / AIDS Cohort Study, and it is exempt from the research ethics review as it does not involve additional data collection or invasive sample collection.

## RESULTS

### Demographics

**Figure 1.**
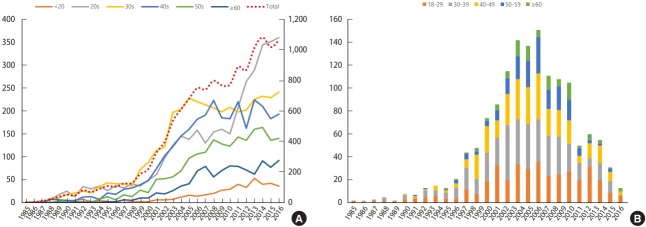
Annual new HIV infections (A: new infections in Korea1 ; B: new infections in Korea HIV/AIDS Cohort Study) by age. HIV, human immunodeficiency virus. ^1^ From Korea Centers for Disease Control. Korea notification of HIV/AIDS in 2017 [Internet] [15].

According to the HIV/AIDS notification in Korea by KCDC, the total number of patients with HIV infection have increased since 2000, and especially since 2010, the number of young adults with HIV infection in their 20s has rapidly increased. Most patients who participated in the Korea HIV/AIDS cohort study were diagnosed with HIV infection between 2006 and 2008, and most of them were in their 30s and 40s (Figure 1). There were 1,345 men (93.3%) and 97 women (6.7%) with HIV infection. At the time of diagnosis, 53.4% of the participants were single, but the number slightly decreased to 52.3% at the time of the baseline survey. The number of those who were married or living together decreased from 25.2% to 24.5%. Moreover, the number of divorced, separated, and widowed individuals increased from 8.7% at the time of diagnosis to 10.6% at the time of the baseline survey. For the transmission route, sexual contact accounts for 94.4%: homosexual (34.2%), bisexual (26.2%), and heterosexual (34.0%). For the place of transmission, 61.6% of the participants were from Korea, 11.2% were from other countries, and 27.2% did not disclose their location. Approximately 805 (55.8%) of the participants reported having symptoms of acute HIV infection within 1 year of HIV diagnosis (Table 1).

### Psychological factors

**Figure 2.**
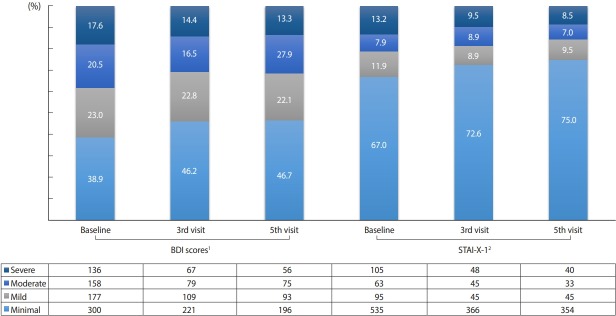
Depression and anxiety status of patients with HIV infection. Values are presented as number. HIV, human immunodeficiency virus; BDI, Beck’s Depression Inventory; STAI-X-1, State-Trait Anxiety Inventory. ^1^ Minimal: <10; mild: 10-15; moderate: 16-23; severe: >-24. ^2^ Minimal: <52; mild: 52-56; moderate: 57-61; severe: >-62.

There were 771 (53.4%) and 798 (55.3%) who had valid scores in the self-reporting of the baseline survey. At the time of the baseline survey, 17.6% and 13.2% of the respondents had severe depression and anxiety, respectively, but after 2 years and 6 months, the proportion decreased to 13.3% and 8.5%, respectively (Figure 2).

### Antiretroviral therapy

Of the total participants, 1,285 (89.1%) participants received ART. The most common types of initial treatment were as follows: two nucleoside reverse transcriptase inhibitors and protease inhibitors (n=716, 55.7%), one nucleoside reverse transcriptase inhibitors and one non-nucleoside reverse transcriptase inhibitor (n=372, 28.9%), two nucleoside reverse transcriptase inhibitors and integrase inhibitors (n=138, 10.7%), and others (n=59, 4.6%).

In the ART adherence survey, 35.0% of patients failed to take their medication on time, and 13.5% skipped the medication once a month. Approximately 91.1% did not adhere to the medication due to side effects, followed by 5.3% who had a fear of revealing diagnosis to others.

The CD4 T cell count was 59.0%, 43.8%, and 45.0%, and the amount of the HIV RNA was 55.4%, 40.8%, and 41.9% in immunoassay within 3 months based on the time of diagnosis, the time of initial treatment start, and the time of initial treatment finish, respectively. The median CD4 T cell count at diagnosis was 229.5 (IQR, 85.5 to 360.5), decreased to 179 (IQR, 60 to 279) at the start of the initial treatment and then recovered to 331 (IQR, 185 to 465) the end of the initial treatment. The HIV viral load was decreased from 63,207.5 copies/mL at diagnosis and 77,100 copies/mL before treatment to 41 copies/mL after the treatment according to the changes in the CD4 T cell count (Table 2).

### Prevalence of AIDS-related/defining and opportunistic infectious diseases

Of 1,442 participants, 240 (16.6%) were diagnosed with HIV-related tuberculosis (TB), and 135 (9.4%) were diagnosed with HIV-related syphilis. The prevalence rates were 65 (4.5%) for oropharyngeal candidiasis, 53 (3.7%) for pneumococcal pneumonia, and 39 (2.7%) for cytomegalovirus infection. Regarding the question of the infectivity of lifelong opportunistic diseases, 386 (26.7%) participants reported that they had been diagnosed with pneumocystis pneumonia, and 155 (10.7%) had been diagnosed with candidiasis (Table 3) currently or previously (including pre-HIV infection diagnosis).

### Prevalence of chronic and other diseases

**Figure 3.**
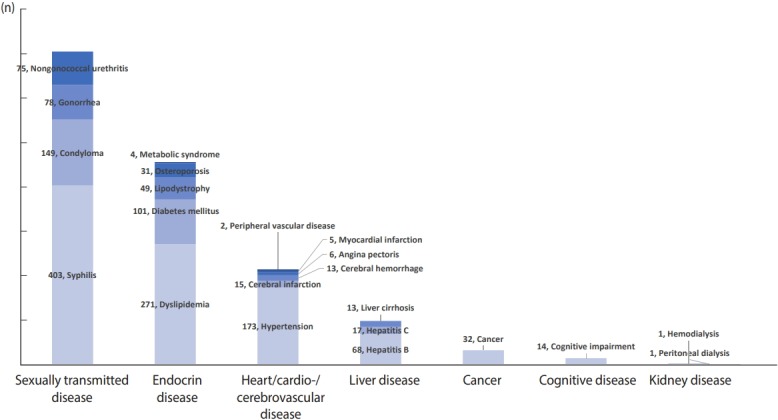
Prevalence of non-AIDS-related diseases. AIDS, acquired immune deficiency syndrome.

The results of the diagnosis of chronic and other diseases showed that the most common disease was syphilis (n=403, 27.9%), followed by dyslipidemia as endocrine disease (n=271, 18.8%), hypertension as cardiovascular disease (n=173, 12.0%), condyloma as sexually transmitted diseases (n=149, 10.3%), and diabetes as endocrine disease (n=101, 7.0%) (Figure 3).

### Reason for termination

Of 1,442 participants in this study, 509 (35.3%) terminated participation in the study due to various reasons. There were 152 dropouts (29.9%), 95 consent withdrawals (18.7%), and 84 deaths (16.5%). Additionally, 55 patients (10.8%) could not complete the study because of poor health, etc. Of 84 deaths, 15 (17.9%) deaths were related to AIDS, and 7 (8.3%) of the others were identified as AIDS non related deaths (Table 4).

## DISCUSSION

Unlike those in other infectious diseases, symptoms of HIV infection are difficult to recognize early, it has a natural course of diseases that causes AIDS to develop slowly as it deteriorates after about 2 to 15 years. For such reasons, it is difficult to recognize the condition such as the exact transmission route at the time of infection. In a report on the population with HIV infection by region states, in the Asia-Pacific region including Korea, there are 5.2 million individuals with HIV infection as of 2017, and approximately 280,000 individuals (16% of the newly infected patients worldwide in 2017) were predicted to be newly infected [2]. A study in the USA predicted that 21% of the infected population were undiagnosed [21]. In Korea, there are also difficulties in evaluating the exact number of the patients with infection because most cases are accidentally found during regular examination or diagnosis for other health issues [15] rather than during voluntary HIV screening test. In 2017, there were 1,191 patients with new HIV infection, and 12,320 were reported for the cumulative infection status of domestic HIV/AIDS in Korea. Approximately 11.7% of these patients were registered in the study, this is large rather than the other HIV studies [22-24]. The cohort data showed similar sex ratio (approximately 93% of men and 7% of women) and the most common transmission route (sexual behavior) with the HIV/AIDS notification report. This study found that 94% of infections were caused by sexual contact. Approximately 60% of these infections were contacted with same-sex or bi-sex, and this was higher than the result of the survey by expert in the public health center [15], which found that, of 11,604 respondents with infection, approximately 42.6% obtained the infection through heterosexual behavior, and 33% through homosexual or bisexual behavior. As mentioned in previous studies, building rapport with medical professionals through institution visits for treatment for a long period rather than relying on the epidemiological report, which is designed for a one-time investigation to report infections, might have attributed to the results [24].

Several previous national and global studies have found that individuals with HIV/AIDS are concerned about exposure of the diagnosis due to social prejudice. In fact, this social stigma influences treatment adherence [25] and increases the incidence rate of mental health issues, including anxiety [26], depression [27-29], suicidal ideation [30], and quality of life [31] compared to the population without infection [32-39]. This study showed the consistent result that the elderly population had a higher depression score (p=0.004), and the anxiety test, performed within 6 months of the time of HIV infection diagnosis, showed a high score (p= 0.007). Although the survival rate has been improved by the development of therapeutic medications, patients are still more likely to develop severe depression during long-term treatment and severe anxiety soon after diagnosis due to social stigma.

UNAIDS has developed a strategy to combat AIDS: 90-90-90 (90% of patients with HIV infection are aware of their diseases, 90% are using medication, and 90% remain to have viral suppression) and continuously monitor the progress. According to reports, 75% of individuals with infection worldwide know their infection status, 79% have been treated, and 81% will remain in the virus suppression state through treatment [1]. This study showed a high treatment rate of approximately 90%. In some cases, combinations of medications other than the recommended therapeutic agents (2NRTI+PI, 2NRTI+NNRTI, 2NRTI+INI) were prescribed during the initial treatment. This might be due to the challenges in accurate investigation in the case of the first treatment experience in another hospital. In this study, 260 patients (20.2% of the total treatment experience, 94.9% of the 274 subjects who had the test result from both beginning and end of the initial treatment) had lower amount of virus compared to that in the pre-treatment. Of 539 patients who had the HIV viral load results immediately after initial treatment, 281 (52.1%) had <50 copies/mL of HIV, which means that approximately 52% of individuals with infection had successful viral suppression through initial treatment.

Previously, various coinfection diseases such as opportunistic infections and AIDS-related/defining diseases were the main indicators of increased mortality rate in HIV infected patients. Recently, the number of patients with coinfection diseases with HIV has been decreasing due to development of various ARTs. The participants in this study still showed high rates of simultaneous infections of syphilis, TB (including mycobacterium TB and non-mycobacterium TB), and pneumocystis pneumonia. Based on the facts that the mean CD4 T cell count at diagnosis was low and 372 (43.7%) of 852 participants had a CD4 T cell count at diagnosis of <200, it can be deduced that the HIV infection was diagnosed late [40]. Studies also revealed that a late HIV diagnosis is likely to cause an active spread of infection to others, as the amount of the virus is not suppressed and missing critical period could lead to less effective treatment, and an increased risk of AIDS and death [41]. Comparing 345 participants who had both CD4 T cell at the time of diagnosis and HIV viral load at the start and end of the initial treatment, of 184 who had the serological AIDS (<200 of CD4 T cells counts) at the time of diagnosis, only 41.9% had viral suppression under 50 copies/mL after initial treatment. However, 57.8% of 161 participants with >200 CD4 T cells counts at the time of diagnosis successfully inhibited the amount of HIV viral after the initial treatment (p=0.003). These results suggest that strategies are required for early diagnosis and timely treatment for efficient outcome of treatment and reduction in the simultaneous incidence of AIDS-related/defining diseases.

An overwhelming number of patients reported that they skipped medication due to the side effects, and this was consistent with the results of previous domestic studies that pointed out that the main reason for changing treatment medication was the reason of side effects [42]. The side effects of medication can influence treatment adherence, and low adherence is associated with treatment failure; therefore, ongoing follow-up and related studies are needed.

It is important for HIV infected patients to receive medical attention to prevent further HIV transmission because it becomes more challenging to prevent the spread of HIV infection if the virus is not successfully suppressed due to low treatment adherence even if a patient is aware of HIV infection but misses the critical period of treatment. As of 2019, the repeat rate was 64.7%, and the adjusted repeat rate was 68.7%, excluding those who were unable to participate due to death or loss to follow-up:

$$\mathrm{Repeat}\;\mathrm{rate}\;=\frac{the\;No.\;of\;participants\;who\;are\;currently\;in\;the\;study\;except\;for\;those\;who\;terminated\;participating\;study}{the\;No.\;of\;participants\;who\;initially\;registered\;for\;the\;HIV/AIDS\;cohort\;study\;in\;Korea}$$

$$\mathrm{Adjusted}\;\mathrm{repeat}\;\mathrm{rate}\;=\frac{the\;No.\;of\;participants\;who\;are\;currently\;in\;the\;study\;except\;for\;those\;who\;terminated\;participating\;study}{\left(the\;No.\;of\;participants\;who\;initially\;registered\;for\;the\;HIV/AIDS\;cohort\;study\;in\;Korea\;–\;the\;No.\;of\;deaths\right)}$$

This study aims to maintain a repeat rate of ≥80%, except for the terminator. In the future, it is necessary to identify the causes that reduce the reinvestigation rate. In addition, survival analysis can be conducted by investigating the causes and date of death, but in this study, the data that contained the information about death could not be insufficient, causes related to most deaths were missing (73.8% of deaths). In the future, it is necessary to reconfirm the medical records for the cause of death or repeat survey using HIV notification data and study the causes or the rate of non-AIDS related deaths, which has recently increased.

To prevent and manage HIV/AIDS at the national level, early diagnosis and appropriate treatment are crucial to prevent the spread of HIV infection and ensure a healthy life for patients. This study provides data that identify the characteristics of 10% of individuals with HIV infection using the only multicenter HIV/AIDS cohort study data in Korea. However, it has limitations that most participants were diagnosed between 2006 and 2008 and the average age at the time of the cohort registration was approximately 40 years, indicating that there is an insufficient number of newly infected patients and young adults. In the future, the recruitment of newly infected young adults is suggested to continue to observe the changing clinical epidemiological indicators of patients with infection and identify the causes of death to develop appropriate domestic medical guidelines.

## Figures and Tables

**Table 1.** General characteristics of research participants

Variables	n (%)
Gender	
Men	1,345 (93.3)
Women	97 (6.7)
Marital status at HIV diagnosis	
Single	770 (53.4)
Married/living together	364 (25.2)
Separated/divorced/widowed	125 (8.7)
Others	141 (9.8)
Missing	42 (2.9)
Marital status at baseline	
Single	754 (52.3)
Married/living together	353 (24.5)
Separated/divorced/widowed	153 (10.6)
Others	140 (9.7)
Missing	42 (2.9)
Acute symptom of HIV	
Yes	805 (55.8)
No	578 (40.1)
Unknown	43 (3.0)
Missing	16 (1.1)
Transmission mode	
Homosexual	493 (34.2)
Bisexual	378 (26.2)
Heterosexual	491 (34.0)
Transfusion/blood products	5 (0.3)
Injected drug use	1 (0.1)
Vertical transmission	0 (0.0)
Others	5 (0.3)
Unknown/no answer	70 (4.9)
Transmission place	
Korea	889 (61.6)
Abroad	161 (11.2)
Unknown	392 (27.2)

HIV, human immunodeficiency virus.

**Table 2.** Characteristics of antiretroviral therapy (ART)

Characteristics	n (%) or median [IQR]
ART	
Yes	1,285 (89.1)
No	157 (10.9)
Initial ART regimen	
2 NRTI+PI	716 (55.7)
2 NRTI+NNRTI	372 (28.9)
2 NRTI+INI	138 (10.7)
Others^1^	57 (4.4)
Unknown	2 (0.2)
Frequency of skipping antiretroviral drugs (multiple responses)	
Never once	2,225 (56.6)
Once a month	532 (13.5)
Once a week	217 (5.5)
Once in 2 weeks	208 (5.3)
More than twice a week	118 (3.0)
Daily	33 (0.8)
Others	269 (6.8)
Missing	328 (8.4)
Cause of skipping ART (multiple responses)	
Side effects of antiretroviral drugs	14,318 (91.1)
Fear of exposure of diagnosis	834 (5.3)
Low socioeconomic status	71 (0.4)
Excessive number of pills	45 (0.3)
Not eating a meal	58 (0.3)
Simply forgot	15 (0.1)
Misinformation about ART	11 (0.1)
Others	9 (0.1)
Missing	359 (2.3)
CD4 T cell count (within 3 mo)	
At HIV diagnosis	852 (59.0)/229.5 [85.5-360.5]
At the start of initial ART	563 (43.8)/179 [60-279]
At finish of initial ART	559 (45.0)/331 [185-465]
HIV RNA (within 3 mo)	
At HIV diagnosis	800 (55.4)/63,207.5 [14,731.5-224,834.0]
At the start of initial ART	524 (40.8)/77,100 [20,200-240,000]
At finish of initial ART	539 (41.9)/41 [20-342]

IQR, interquartile range; NRTI, nucleoside reverse transcriptase inhibitor; PI, protease inhibitor; NNRTI, non-nucleoside reverse transcriptase inhibitor; INI, integrase inhibitor; HIV, human immunodeficiency virus.

1 Others: NRTI, 2 NRTI, NRTI+PI, PI, NRTI+NNRTI, 3 NRTI+PI, NRTI+INI+PI, NRTI+NNRTI+PI, 3 NRTI, INI, NNRTI+PI.

**Table 3.** Characteristics of AIDS-related diseases (including opportunistic infections)

Characteristics	n (%) (n=1,442)^1^
AIDS-related/defining diseases^2^	
Tuberculosis (mycobacterium, non-mycobacterium)	240 (16.6)
Syphilis	135 (9.4)
Candidiasis, oropharyngeal (thrush)	65 (4.5)
Pneumocystis carinii pneumonia	53 (3.7)
Cytomegalovirus disease	39 (2.7)
Herpes zoster	28 (1.9)
Candidiasis, esophageal	22 (1.5)
Herpes simplex	20 (1.4)
Herpes simplex virus: bronchitis, pneumonia, or esophagitis	20 (1.4)
Peripheral neuropathy	9 (0.6)
HIV-related encephalopathy	7 (0.5)
Wasting syndrome due to HIV	6 (0.4)
Kaposi’s sarcoma	5 (0.3)
Idiopathic thrombocytopenic purpura	4 (0.3)
Burkitt’s lymphoma	4 (0.3)
Pneumonia (recurrent)	4 (0.3)
Candidiasis, vulvovaginal	3 (0.2)
Cryptococcosis, extrapulmonary	2 (0.1)
Cryptosporidiosis, chronic intestinal (>1 mo)	2 (0.1)
Immunoblastic lymphoma	2 (0.1)
Progressive multifocal leukoencephalopathy	1 (0.1)
Toxoplasmosis of brain	1 (0.1)
Cervical dysplasia (moderate or severe)	1 (0.1)
Opportunistic infection^3^	
Pneumocystis carinii pneumonia	386 (26.7)
Candida	155 (10.7)
Cytomegalovirus	76 (5.3)
Cryptococcus neoformans	8 (0.6)
Toxoplasma	4 (0.3)
Salmonella	2 (0.1)

AIDS, acquired immune deficiency syndrome; HIV, human immunodeficiency virus.

1 Total prevalence rate.

2 Diagnosis since a year before HIV infection diagnosis to survey date.

3 A lifelong diagnosis experience regardless of the time of HIV diagnosis; No frequency of occurrence of bacillary angiomatosis, listeriosis, pelvic inflammatory disease, candidiasis of bronchi, trachea, or lungs, coccidiodomycosis, disseminated or extrapulmonary, isosporiasis, chronic intestinal (>1 month duration), herpes simplex virus with chronic ulcer(s) (>1 month duration), histoplasmosis, disseminated or extrapulmonary, salmonella septicemia (recurrent), cervical cancer (invasive), primary lymphoma of the brain.

**Table 4.** Characteristics of termination survey

Variables	n (%)
Termination rate	509 (35.3)
Cause of termination	
Loss to follow up (2 yr)	152 (29.9)
Withdraw consent	95 (18.7)
Death	84 (16.5)
Withdrawal of the hospital’s research	70 (13.8)
Changing hospital	52 (10.2)
Immigration	1 (0.2)
Others	55 (10.8)
Cause of death	
AIDS-related death	15 (17.9)
Non-AIDS-related death	7 (8.3)
Unknown/missing	62 (73.8)

AIDS, acquired immune deficiency syndrome.

## SUPPLEMENTARY MATERIALS

Korean version is available at http://www.e-epih.org/.
