# Supplementary material for: Epidemiological characteristics of HIV infected Korean: Korea HIV/AIDS Cohort Study
Source: Epidemiol Health. 2019 Sep 3;41:e2019037. doi: 10.4178/epih.e2019037 (PMC6815876; doi:10.4178/epih.e2019037)
Supplement: Supplementary file 1 [file epih-41-e2019037-supplementary.pdf]

Volume: 41, Article ID: e2019037
https://doi.org/10.4178/epih.e2019037

국내 HIV/AIDS 감염인의 역학적 특성: 한국 HIV/AIDS 코호트 연구

Epidemiological characteristics of HIV infected Korean: Korea HIV/AIDS Cohort Study

Clinical and epidemiological characteristics of people living with HIV/AIDS (PLWHA) in Korea

Authorship:

First author: Yunsu Choi[1), 2)]

Corresponding author: Bo Youl Choi[1), 2)]

Co-author:

Soo Min Kim[2), 3)], Sang il Kim[4)], June Kim[5)], Jun Young Choi[5)], Shin-Woo Kim[6)], Joon Young Song[7)], Youn Jeong Kim[4)], Dae Won Park[8)], HYO YOUL KIM[9)], Hee-Jung Choi[10)], Mee-Kyung Kee[11)], YoungHyun Shin[11)], Myeongsu Yoo[11)]

1) Department of Preventive Medicine, College of Medicine, Hanyang University, Seoul, Korea
2) Institute for Health and Society, Hanyang University, Seoul, Korea
3) Department of Applied Statistics, College of Business and Economic, Yonsei University, Seoul, Korea
4) Division of Infectious Disease, Department of Internal Medicine, Seoul St. Mary's Hospital, College of Medicine, The Catholic University of Korea, Seoul, Korea
5) Department of Internal Medicine and AIDS Research Institute, Yonsei University College of Medicine, Seoul, Republic of Korea
6) Department of Internal Medicine, School of Medicine, Kyungpook National University, Daegu, Korea
7) Division of Infectious Diseases, Department of Internal Medicine, Korea University College of Medicine, Seoul, Korea.
8) Department of Internal Medicine, Yonsei University Wonju College of Medicine, Wonju, Republic of Korea
9) Department of Internal Medicine, Korea University College of Medicine, Seoul, Korea

10) Department of Internal Medicine, Ewha Womans University College of Medicine, Seoul, Republic of Korea
11) Division of Viral Disease Research Center for Infectious Disease Research, Korea National Institute of Health, Cheongju-si, Korea

Corresponding author:

Dr. Bo Youl Choi / Department of Preventive Medicine, Hanyang University College of Medicine
E-mail: bychoi@hanyang.ac.kr

Acknowledgements:

이 연구는 질병관리본부 만성 감염 질환 코호트 사업(한국 HIV/AIDS 코호트 연구)으로 지원받아 수행되었습니다(2016-E51003-02).

Ethics statement:

이 연구의 수행 목적은 한국 HIV/AIDS 코호트 연구와 동일하며, 연구를 위해 대상자에게 추가 자료를 수집하거나 침습적 검체 수집이 이뤄지지 않아 연구윤리심의 면제 대상입니다.

초록

목적

전 세계적으로 HIV 신규 감염인이 더 이상 발생하지 않기 위한 노력이 지속되고 있다. 실제로 1995 년 이후 전 세계 HIV 신규 감염인의 수는 꾸준히 감소하고 있다. 근거 기반의 질병 관리를 위해서는 각 국의 감염인의 특성을 파악하는 것이 중요한데, 국내에서는 전국적인 자료를 활용한 감염인의 역학적 특성을 파악한 연구가 없었다.

방법

한국 HIV/AIDS 코호트 연구는 전국 21 개의 대학병원에 방문한 내국인 HIV 감염인 1,442 명(2017 년 기준 국내 내국인 감염인의 약 11%)에 대한 역학적 특성을 파악하고자 한다. 2006 년 12 월부터 2016 년 12 월까지 조사에 참여한 1,442 명의 대상자의 조사 기록 중 1~5 회차(기반 조사 이후 반복 4 회차 조사) 자료를 사용하였다.

결과

남성이 전체의 93%였고, 전체 대상자의 약 56%에 해당하는 감염인이 급성 감염기 증상을 겪었다고 응답했다. 감염경로에 따르면 성 접촉으로 인한 감염이 전체의 94.4%를 차지했고, 이 중 60.4%가 동성 혹은 양성 간 성접촉으로 인해 감염되었다. 감염 장소는 국내가 61.6%, 국외가 11.2%였고, 대상자는 조사를 반복하면서 우울 및 불안의 점수가 감소하는 경향을 보였다. 전체 대상자의 89.1%가 치료를 경험하였다. 최초 치료 약제는 권고안에 따른 경우가 전체의 95.3%였고, 약물 복용을 거르는 경험이 있는지 여부에서 한번도 없었다고 응답한 경우가 56.6%였고, 약물 복용을 거르는 주요 사유로는 약제의 부작용이 91.1%로 가장 높게 나타났다. 면역 상태 결과에 따르면 국내 감염인은 진단시점 CD4 세포 수의 중앙값이 229.5 였고, 치료 시작 시점 179 로 감소하였다가, 치료

후 331 로 개선되었다. 최초 항레트로바이러스 치료 후 HIV 바이러스 양의 중앙 값이 41 이었다. HIV 관련/정의질환으로는 결핵과 매독이 각각 16.6%, 9.4%로, 기회감염에서는 주 폐포자충 폐렴이 26.7%로 높은 유병률을 보였다. 이 외의 과거질환력에서는 성매개 감염병이 가장 높은 유병률을 보였고, 이어서 내분비계 질환의 유병률이 높았다. 종결 사유로는 중도탈락 대상자가 29.9%로 가장 높았고, 동의를 철회한 대상자가 18.7%였다.

결론

이 연구에 따르면 국내 감염인은 대부분 성 접촉으로 인해 감염되었고, 감염인의 약 90%가 치료를 경험하였다. 하지만 여전히 결핵, 매독, 주폐포자충 폐렴 등과 같은 HIV 관련 질환의 유병률이 높았고, 치료를 통해 수명이 연장되어 노화 혹은 치료 약제 부작용과 관련된 HIV 비 관련 만성 질환의 유병률도 높았다. 향후 감염인의 HIV/AIDS 비 관련 질환에 대한 연구 및 사망 원인 등에 대한 후속 연구가 필요하겠다.

1. 서론

유엔 에이즈 합동 계획(UNAIDS; The Joint United Nations Programme on Acquired Immune Deficiency Syndrome)은 2030년까지 HIV (Human Immunodeficiency Virus) 전염 퇴치를 선언했고, 2020년까지 전 세계 신규 감염인 수가 50만 명 이하로 줄어드는 것을 목표로 하고 있다 [1]. 국가별로 감염인의 역학적 특성을 고려하여 지속 가능한 개발을 수립 및 수행하고 있으며, 실제로 HIV 신규 감염인의 수는 적극적인 개발 계획을 통해 1995년 이후 꾸준히 감소하였다 [2]. HIV/AIDS를 처음 인식한 1980년대에는 급성 감염 증상과 각종 기회감염 및 관련/정의 질환 등의 HIV 관련 질환으로 인한 사망률이 높았던 [3] 반면, 1997년 지도부딘(zidovudine)을 시작으로 각종 항레트로바이러스 치료제(ART; antiretroviral therapy)가 개발되면서 바이러스 억제와 면역 수준 향상 등의 치료 효과를 통해 AIDS 관련 사망률이 감소하고 있다. 하지만 장기간 약물치료에 따른 신장, 간 등의 대사성 질환의 발생이 증가, 그리고 HIV 감염 후 생존 기간이 증가함에 따라 노화로 인한 심혈관질환 및 폐 질환 등의 만성질환과 HIV 비관련 암으로 인한 사망이 증가하는 등 HIV/AIDS 질병의 자연사가 변화하고 있다 [4-9].

변화하는 HIV 자연사를 파악하기 위해 해외 각국에서는 HIV 코호트 연구를 수행 중이다. 최초의 HIV 코호트 연구는 1983년 미국에서 시작된 다기관 AIDS 코호트 연구(MACS; Multicenter AIDS Cohort Study)인데, 현재까지도 대상자를 모집하여 활발한 연구를 수행 중이며 HIV/AIDS 진료 지침과 자연사에 대해 많은 연구가 진행되었다. 이 연구의 주 대상자는 HIV 감염의 고위험군으로 동성 간 성 접촉을 경험한 남성(MSM; Men who Sex with Men)이며, 혈청학적으로 HIV 음성인 대상자여도 연구 참여가 가능해 고위험군에서의 HIV/AIDS 발생 등 혈청학적 변화에 대한 기전을 확인할 수 있다는 장점이 있다 [10]. 이후 HIV 감염인이 계속해서 증가하며 스위스 [11], 영국 [12], 덴마크 [13] 등에서 다양한 HIV 코호트가 구축되어 활발한 연구가 진행되었다. 하지만 코호트별로 연구 대상자의 연구 참여 기준이 상이하고, 기존 연구에서 언급된 바와 같이 인종과 성별이 HIV-1 질병 진행률에 영향을 미치기 때문에

각국에서 진료 및 치료 지침을 마련할 때에는 해당 국가의 특성을 고려해 조정될 필요가 있다 [14].

국내에서는 1985년 외국인 1명, 내국인 1명의 최초 감염 2인을 시작으로 1995년 처음으로 연간 100명이 넘는 감염인이 발생하였고, 이후로 꾸준히 증가하여 2013년 이후에는 매년 1,100명 이상의 신규 감염인이 HIV 진단을 받았다 [15]. 우리나라는 HIV 감염인의 건강 유지와 타인으로의 전파 방지를 위해 국민건강보험 보장 대상자 가운데 양성 확진 이후 국가 시스템에 등록한 감염인에 대해 HIV 관련 의료비를 건강보험에서 90%, 국가에서 본인부담금의 10%를 지원하고 있다 [16]. 이는 경제적인 이유로 치료를 받지 못하는 감염인이 없도록 하기 위함이다.

국가별 HIV 감염인을 위한 맞춤형 진료 지침 및 치료 전략을 구축하기 위해 국내 감염인에 대한 정확한 특성을 파악하는 것이 매우 중요하다. 이에 국내 유일의 HIV/AIDS 코호트 자료를 이용하여 국내 HIV 감염인의 역학적 특성을 파악하여 근거자료로 사용하고자 한다.

2. 연구방법

2.1. 연구 자료 및 분석 방법

한국 HIV/AIDS 코호트 연구를 통해 2006년 12월부터 2016년 12월까지 기반조사부터 반복 4회차(5회 방문)까지 자료로 총 1,442명의 조사서 5,795건의 자료를 사용하였다. 현재까지 사망, 전원/이주, 동의 철회 등의 사유로 연구에 참여하지 않는 대상 509명을 제외하고, 현재까지 연구에 참여 중인 대상자는 933명이다 [17].

모든 결과는 결측값을 포함하여 기술 통계량으로 제시하였다. 자료 구축 및 통계적 분석은 SAS Enterprise guide 7.1 ver. 통해 산출되었다. 범주형 자료에 대해서는 빈도와 백분율을 제시하였고, 비정규분포를 나타내는 혈액검사결과에 대해서는 중앙값(median)과 사분범위(interquartile range, IQR)로 제시하였다.

2.2. 자료수집방법

반복 조사는 6개월마다 시행되고, 일부 문항의 종류에 따라 단기간에 변화하지 않는 문항의 조사 주기는 12개월로 정한다. 기반 조사 시점에는 평생 혹은 HIV 진단을 기준으로 발생한 사건을 조사하고, 반복 조사 시에는 지난 조사 이후 모든 기록을 조사하여 코호트 연구 자료를 구축하였다. 인구학적 특성에 해당하는 성별, 연령, 결혼 상태와 감염경로는 자가 작성 설문조사로 수집하였다. 정신 심리적 요인 척도 검사도 자가 작성 설문조사로 이뤄졌는데, 우울 검사 도구는 총 21개 문항의 Beck의 우울증 척도 검사-한국어판 (Beck's Depression Inventory: BDI), 총 20개 문항의 불안 검사 도구는 상태 불안 척도 (State-Trait Anxiety Inventory: STAI-X-1)로 조사되었다. 각 설문조사결과에 따른 점수를 4개 군으로 구분하여 증상의 중증도를 정의하였다 [18-20]. 항레트로바이러스 치료 및 순응도, 만성질환 및 기타 관련 질환은 환자 면담과 의무기록을 통해 조사하였다. 채혈 검사 결과는 각 정의된 시점을 기준으로 3개월 이전까지를 동일한 시점으로 정의한다. AIDS 관련/정의 질환은 HIV 진단일 기준 1년 전부터 조사 시점까지의 동안 전문의로부터 진단 여부를 조사하였고, 기회 감염은 HIV 진단과 관련없이 평생 진단 여부를 조사하였다. 종결조사서는 조사자 면담을 통해 진행하거나, 중도탈락대상자에 대해서는 의무기록 혹은 국가 신고 자료를 이용하여 사망 여부를 추적조사 하였다.

3. 결과

3.1. 인구학적 특성

국내 HIV/AIDS 내국인 신고 현황에 따르면 2000년 이후 전반적으로 상승하는 형태를 보였고, 특히 2010년 이후 20대의 젊은 성인이 급격히 증가하는 것을 확인할 수 있었다. 한국 HIV/AIDS 코호트 연구에 참여한 대부분 대상자는 2006년부터 2008년 사이에 HIV 진단을 받았고, 연령 분포는 30~40대의 비율이 가장 높았다(Figure 1), 남성은 1,345명(93.3%), 여성은 97명(6.7%)이었다. HIV 진단 시점 결혼상태로는 미혼인 대상자가 전체의 53.4%였는데, 기반 조사 시점에는 52.3%로 소폭 감소하였고, 결혼 혹은 동거 중인 대상자는 진단 시점 25.2%에서 기반 조사 시점 24.5%로 감소하였다. 이혼, 별거, 사별의 경우 진단 시점 8.7%인데 반해

Volume: 41, Article ID: e2019037
https://doi.org/10.4178/epih.e2019037

기반 조사 시점에는 10.6%로 증가하였다. 감염 경로에서는 동성 간 성접촉을 통한 감염이 34.2%로 가장 높았고, 양성 간 성접촉이 26.2%, 이성 간 성접촉이 34.0%로 전체 감염경로 중 성접촉으로 인한 감염이 94.4%에 달하였다. 감염 장소는 국내가 전체의 61.6%, 해외에서의 감염이 11.2%, 정확히 알 수 없다고 응답한 경우가 27.2%였다. 805명(56%)의 대상자가 진단일 기준 1년 이내 HIV 급성기 감염 증상을 경험하였다고 응답했다(Table 1).

3.2. 정신 심리적 요인

기반 조사 시점 자가작성 설문에 응답하여 유효한 점수를 보유한 대상은 각각 771명(53.4%), 798명(55.3%)이다. 기반 조사 당시 심각한 수준의 우울감과 불안감을 경험한 대상은 각각 응답자의 17.6%, 13.2%였으나, 약 2년 6개월이 지난 후의 반복조사에서는 각각 13.3%, 8.5%로 감소하였다(Figure 2).

3.3. 항레트로바이러스 치료

전체 대상자 중 1,285명(89.1%)이 항레트로바이러스 치료를 경험하였고, 치료를 받은 대상의 최초 치료제 종류는 2개의 뉴클레오사이드 역전사효소 억제제와 단백분해효소 억제제(716명, 55.7%), 1개의 뉴클레오사이드 역전사효소 억제제와 1개의 비 뉴클레오사이드 역전사효소 억제제 (372명, 28.9%), 그리고 2개의 뉴클레오시드 역전사 효소 억제제와 통합효소 억제제(138명, 10.7%) 순으로 높았다. 이 외에 59명(4.8%) 이 서로 다른 약제를 이용한 약물 조합을 사용하였다.

약물 순응도 조사에서 약을 제때 먹지 못한 경우가 전체의 35%였고, 한 달에 한 번 정도 거르는 경우가 13.5%에 달했다. 약물 거르는 사유로는 약제의 부작용에 따른 경우가 91.1%로 가장 높았고, 타인에게 노출될 것이 두려운 경우가 5.3%로 그 뒤를 이었다.

진단 시점, 최초 치료 시작 시점 종료 시점을 기준으로 3개월 이내에 검사한 면역 검사 결괏값은 CD4 T 세포 수가 각각 59.0%, 43.8%, 45.0%의 응답률을 보였고, HIV 바이러스양이 55.4%, 40.8%, 41.9%의 응답률을 보였다. 진단 시점 CD4 T 세포 수의 중앙값은 229.5개(IQR;

85.5-360.5)이고, 최초 치료 시작 시점 179개(60-279)에서 치료 후 331개(185-465)로 중앙값 이 증가하였다. HIV 바이러스양은 CD4 변화에 따라 진단 시점 63,207.5 copies/ml, 치료 시작 전 77,100 copies/ml 그리고 치료 후 41 copies/ml로 감소하였다(Table 2).

3.4. AIDS 관련/정의 질환 및 기회 감염 질환 유병률

전체 1,442명의 대상자 가운데 240명(16.6%)은 HIV 진단과 관련된 결핵을 진단받았고, 135명(9.4%)은 HIV 관련 매독을 진단받았다. 이 외에 구인두 칸디다증이 65명(4.5%), 주 폐포자충 폐렴이 53명(3.7%), 그리고 거대 세포 바이러스 감염증이 39명(2.7%)으로 순으로 유병률이 높았다. 평생 기회 감염 질환의 감염력을 묻는 문항에서는 전체 대상자 가운데 386명(26.7%)이 주 폐포자충 폐렴에 현재 혹은 과거(HIV 진단 이전 포함)에 진단받은 경험이 있고, 155명(10.7%)이 칸디다 증으로 진단받았다(Table 3).

3.5. 만성질환 및 기타 질환 유병률

평생 만성질환 및 기타 질환을 진단 여부를 살펴보면 성매개 감염병의 매독(403명, 27.9%)이 가장 높았고, 내분비계 질환의 이상지질혈증(271명, 18.8%), 심뇌혈관질환의 고혈압(173명, 12.0%), 그리고 성매개감염병의 콘딜로마(149명, 10.3%)와 내분비계 질환의 당뇨(101명, 7.0%)순으로 높은 유병률을 보였다(Figure 3).

3.6. 종결 사유

이 연구의 참여자 1,442명 가운데 509명(35.3%)이 여러 종결 사유로 인해 연구에 더는 참여하지 않는다. 중도탈락자 152명(29.9%), 동의철회자가 95명(18.7%)이었고, 사망자가 84명(16.5%)이었다. 이 외에 연구에 참여할 수 없는 개인의 건강 상태 악화 등을 이유로 연구를 종결한 대상이 55명(10.8%)이었다. 사망자 84명 가운데 15명(17.9%)은 선행 사인이 AIDS와 관련된 사망이었고, 7명(8.3%)은 비 AIDS 관련 사망으로 확인되었다(Table 4).

4. 고찰

Volume: 41, Article ID: e2019037
https://doi.org/10.4178/epih.e2019037

HIV 감염은 다른 감염성 질환과 달리 즉시 감염 사실을 인지하기 어렵고, 약 2~15년에 걸쳐 서서히 면역이 저하되어 AIDS가 발생하는 등의 질병 자연사를 가지고 있다. 이러한 이유로 정확한 감염경로 등의 감염 시 상황을 파악하는 것이 쉽지 않다. HIV 감염인구를 지역별로 보았을 때, 한국이 속한 아시아태평양 지역에는 2017년 기준 약 520만명이 HIV 감염 상태로 살아가고 있고, 2017년 한 해에만 약 28만명(2017년 전 세계 신규감염자의 16%)이 감염되었다고 추정했다 [2]. 미국의 한 연구에서는 실제 감염 인구 중 21%가 진단되지 않은 상태로 살아가고 있다고 예측했다 [21]. 국내 감염 인구 역시 자발적 검사 이외에 기타 검사 및 건강 검진 등의 우연한 기회로 감염 사실을 인지하는 경우가 많아 [15] 정확한 감염자 수를 파악하기 어렵다. 2017년 기준 국내 신규 HIV 감염자는 1,191명이고, 내국인 HIV/AIDS 누적감염현황으로는 12,320명이 보고되었는데, 한국 HIV/AIDS 코호트 연구는 이 중 총 1,442명의 대상자가 이 연구에 등록되어 내국인 신고 감염 인구의 약 11.7%에 달하는 자료로 타 HIV 연구보다 대형 코호트 연구에 속한다 [22-24]. 실제 신고 자료와 동일하게 코호트 자료에서도 남성이 약 93%, 여성이 7%의 비율을 보였고, 감염경로에서도 성접촉으로 인한 감염이 가장 많은 비율을 차지하였다. 이 연구에서는 성접촉으로 인한 감염이 전체의 94%에 해당하고, 이 대상자 중 약 60%의 감염인이 동성 혹은 양성 간 성접촉으로 전파되었다고 나타났는데, 이는 담당 보건소 직원에 의한 설문조사 [15]에서 응답한 11,604명의 감염인 중 약 42.6%가 이성으로 인한 성접촉, 그리고 33%가 동성 혹은 양성 간의 성접촉으로 전파되었다는 보고를 웃도는 결과이다. 기존 연구에서도 언급한 바와 같이 감염 신고를 위해 일회성으로 조사하는 역학 신고 조사와 달리 치료를 위해 오랜 기간 의료기관을 방문하여 라포(rapport)가 형성된 의료진에게 좀 더 솔직하게 응답할 수 있어 얻어진 결과라고 예상된다 [24].

기존의 국내외 많은 연구를 통해 HIV/AIDS 감염인은 사회적 편견으로 인해 질병의 노출을 걱정한다는 것은 밝혀진 바 있다. 실제로 이러한 사회적 낙인은 치료 순응도에도 영향을 미치고 [25], 비감염집단에 비해 불안 [26], 우울 [27-29], 자살 생각 [30], 삶의 질 [31] 등을 포함한 정신건강 문제 [32-39]의 발생률을 높이는 원인이 된다. 실제로 이 연구에서는 조사 당시

Volume: 41, Article ID: e2019037
https://doi.org/10.4178/epih.e2019037

고령인 군에서 우울 점수가 높았고(p=0.004), 불안 검사를 HIV 진단 시점으로부터 6개월 이내에 시행한 경우 불안 점수가 높았다(p=0.007). 치료제의 개발로 생존율이 높아졌으나, 장기간 치료를 시행하며 심한 우울감을 경험하는 비율이 높아지고, 사회적 낙인으로 인해 진단 초기 심한 불안감을 경험하는 것으로 예상된다.

UNAIDS는 AIDS 퇴치를 위한 '90-90-90 (HIV 감염인의 90%가 그들의 감염 상태를 인지하고, 90%가 약물치료를 받으며, 90%가 바이러스 억제 상태를 유지하는 것)' 전략을 수립하여 지속해서 모니터링하고 있다. 보고에 따르면 전 세계의 75%의 대상자가 그들의 감염 상태를 알고, 79%가 치료를 받았으며, 81%의 감염인이 치료를 통해 바이러스 억제 상태를 유지할 것이라고 추정했다 [1]. 이 연구에서는 약 90%의 높은 치료 시행률을 보인다. 최초 치료력에서 권고된 치료 약제(2NRTI+PI, 2NRTI+NNRTI, 2NRTI+INI) 이외의 다른 약제 조합이 처방된 경우가 있었다. 이는 실제 타 병원에서 최초 치료 경험하는 등의 경우에 조사가 정확하게 이뤄질 수 없어 회상 비뚤림이 있었을 것으로 예상한다. 이 연구에서 치료를 통해 치료 전보다 바이러스양이 줄어든 대상은 260명(전체 치료 경험자 중 20.2%, 치료 시작 및 종료 시점 검사 결괏값이 모두 있는 대상 274명 중 94.9%)이였고, 치료 종료 시점 RNA 검사 결괏값이 있는 539명 중 281명(52.1%)이 HIV 바이러스양이 50 copies/ml 미만으로 검출되어 최초 치료를 통해 약 52%의 감염인은 성공적으로 바이러스가 억제되었다.

과거에는 HIV 감염 후 각종 기회감염 및 관련/정의 질환의 동시 감염이 HIV 감염인의 사망률을 높여 주요 관심 지표였다. 최근에는 다양한 항레트로바이러스 치료제가 개발되고, 각종 기회감염 및 관련/정의 질환의 동시 감염이 감소하는 추세이다. 이 연구에서는 여전히 HIV 진단과 관련된 매독과 결핵(항산균, 비결핵 항산균 포함), 그리고 주 폐포자충 폐렴의 동시감염률이 높았다. 이는 대상자의 진단 시점 평균 CD4 T 세포수가 낮고, 진단 시점 CD4 결괏값이 있는 852명의 대상자 중 372명(43.7%)가 진단 시 CD4가 200개 미만으로 면역학적 AIDS에 해당한 것으로 미루어보아, 조기에 HIV 진단이 되지 않았음(late diagnosis)을 추정할 수 있다 [40]. 실제로 HIV 진단이 늦어지면 바이러스 양이 억제되지 않아 타인에게 감염을 확산

Volume: 41, Article ID: e2019037
https://doi.org/10.4178/epih.e2019037

시킬 가능성이 높고, 적절한 시기에 치료를 받지 못해 치료의 효과가 떨어지거나, AIDS 발생 및 사망 위험이 증가한다는 연구 결과가 있는데 [41], 이 연구에서도 진단 시점 CD4와 최초 치료 종료 시점 RNA 결괏값을 모두 가지고 있는 대상자 345명에 대하여 진단 시점 면역 상태와 최초 치료 결과를 비교하였을 때, 진단 시점 면역학적 AIDS (CD4 세포 수 200개 미만)인 대상자 184명 중 최초 치료 후 바이러스양이 50 copies/ml로 감소한 대상자는 전체의 41.9%에 불과한 반면, 진단시점 CD4가 200개 이상인 대상자 161명 중 57.8%가 초치료 후 바이러스 양이 성공적으로 억제되어 진단시점 CD4가 초치료 결과에 유의한 영향을 미치는 것을 알 수 있었다(P=0.003). 이러한 결과를 통해 감염인의 성공적인 치료 결과와 AIDS 관련/정의 질환의 동시 감염 발생을 감소시키기 위해 조기 진단 및 적정 치료 시점에 대한 전략이 필요하다.

약물 복용을 거르는 사유로 약물 부작용이 압도적으로 높은 비율을 보였는데, 기존 국내 연구에서 치료 약제를 변경하는 주된 이유가 약물 부작용이라는 결과와 동일했다 [42]. 약제 부작용은 약물 순응도 저하에 영향을 미치고 낮은 약물 순응도는 치료 실패와 연관 되므로, 이에 대한 지속적인 추적 관찰과 관련 연구가 필요하겠다.

HIV 예방 관리를 위해 감염인이 의료기관의 케어를 받는 것은 중요하다. 실제로 감염인이 감염 사실을 인지하였음에도 적정 치료 시기를 놓치거나, 치료 순응도가 낮아 성공적인 바이러스 억제가 되지 않는다면 타인으로의 전파가 용이해져 HIV 전파 예방이 어렵기 때문이다. 2019년 현재까지 반복 조사율[1]은 64.7%이고, 추적이 불가능해서 조사에 더는 참여하지 못하는 사망자를 제외하여 계산된 보정 반복 조사율[2]은 68.7%이다. 이 연구에는 종결자를 제외하고 80% 이상의 반복 조사율을 목표로 추적 관찰 중이므로 향후 반복 조사율을 낮추는 원인

---

1 $\frac{\text{종결대상을 제외한 현재 유지자 수}}{\text{한국 HIV/AIDS 코호트 등록 대상자}}$

2 $\frac{\text{종결 대상을 제외한 현재 유지자 수}}{\text{(한국 HIV/AIDS 코호트 등록 대상자 수 − 사망자 수)}}$

https://doi.org/10.4178/epih.e2019037

을 파악할 필요가 있다. 또한, 추적조사를 통해 사망 여부와 사망일을 조사하고 있어 생존에 관한 분석을 시행할 수 있으나, 실제로 정확한 사망원인에 대한 자료가 누락되어 추적조사를 통해 사망이 확인된 대상자의 사망원인은 모두 결측값(사망자의 73.8%)으로 처리되어 있다. 향후 사망원인에 대한 의무기록 재확인 혹은 신고자료를 이용한 추적조사를 시행하고, 최근 증가하고 있는 비 AIDS 관련 사망률 등의 사망 원인에 관한 연구를 시행할 필요가 있다.

국가 차원에서의 HIV/AIDS 예방 및 관리를 위해서는 조기에 진단하고, 적절하게 치료를 시작하여 HIV 감염 확산을 사전에 예방하고, 감염인이 보다 건강한 삶을 영위할 수 있도록 해야 한다. 이 연구는 국내 유일의 다기관 HIV/AIDS 코호트 연구로 내국인 감염자의 10%에 해당하는 특성을 확인할 수 있는 자료이다. 하지만 대부분 2006~2008년 사이에 진단되었고, 코호트 입적 시점 연령이 평균 약 40세인 자료로 신규 감염인과 젊은 성인이 부족하다는 한계가 있다. 이에 향후 최근에 감염된 신규 및 젊은 성인 감염인을 추가 모집하여 변화하는 국내 감염인의 임상역· ◌학적 지표에 대하여 지속 관찰하고, 자연사를 파악하여 적정한 국내 진료 지침을 개발해야 한다.

5. 참고문헌

1. Joint United Nations Programme on HIV/AIDS. HIV prevention 2020 road map: accelerating HIV prevention to reduce new infections by 75% [cited 2019 Oct 16]. Available from: https://www.unaids.org/sites/default/files/media_asset/hiv-prevention-2020-road-map_en.pdf.
2. Joint United Nations Programme on HIV/AIDS. 2018 global HIV statistics [cited 2019 Aug 12]. Available from: https://www.unaids.org/sites/default/files/media_asset/UNAIDS_FactSheet_en.pdf.
3. Chorba TL, Holman RC, Clarke MJ, Evatt BL. Effects of HIV infection on age and cause of death for persons with hemophilia A in the United States. Am J Hematol 2001;66:229-240.
4. Antiretroviral Therapy Cohort Collaboration. Causes of death in HIV-1-infected patients treated with antiretroviral therapy, 1996-2006: collaborative analysis of 13 HIV cohort studies. Clin Infect Dis 2010;50:1387-1396.

5. Palella FJ Jr, Baker RK, Moorman AC, Chmiel JS, Wood KC, Brooks JT, et al. Mortality in the highly active antiretroviral therapy era: changing causes of death and disease in the HIV outpatient study. J Acquir Immune Defic Syndr 2006;43:27-34.

6. Weber R, Sabin CA, Friis-Møller N, Reiss P, El-Sadr WM, Kirk O, et al. Liver-related deaths in persons infected with the human immunodeficiency virus: the D:A:D study. Arch Intern Med 2006; 166:1632-1641.

7. Monforte Ad, Abrams D, Pradier C, Weber R, Reiss P, Bonnet F, et al. HIV-induced immunodeficiency and mortality from AIDS-defining and non-AIDS-defining malignancies. AIDS 2008;22: 2143-2153.

8. Reisler RB, Han C, Burman WJ, Tedaldi EM, Neaton JD. Grade 4 events are as important as AIDS events in the era of HAART. J Acquir Immune Defic Syndr 2003;34:379-386.

9. Núñez MJ, Martín-Carbonero L, Moreno V, Valencia E, Garcia-Samaniego J, Castillo JG, et al. Impact of antiretroviral treatment-related toxicities on hospital admissions in HIV-infected patients. AIDS Res Hum Retroviruses 2006;22:825-829.

10. Detels R, Jacobson L, Margolick J, Martinez-Maza O, Muñoz A, Phair J, et al. The multicenter AIDS Cohort Study, 1983 to .... Public Health 2012;126:196-198.

11. Swiss HIV Cohort Study, Schoeni-Affolter F, Ledergerber B, Rickenbach M, Rudin C, Günthard HF, et al. Cohort profile: the Swiss HIV Cohort study. Int J Epidemiol 2010;39:1179-1189.

12. UK Collaborative HIV Cohort Steering Committee. The creation of a large UK-based multicentre cohort of HIV-infected individuals: the UK Collaborative HIV Cohort (UK CHIC) Study. HIV Med 2004:5:115-124.

13. Lohse N, Hansen AB, Jensen-Fangel S, Kronborg G, Kvinesdal B, Pedersen C, et al. Demographics of HIV-1 infection in Denmark: results from the Danish HIV Cohort Study. Scand J Infect Dis 2005;37:338-343.

14. Anastos K, Gange SJ, Lau B, Weiser B, Detels R, Giorgi JV, et al. Association of race and gender with HIV-1 RNA levels and immunologic progression. J Acquir Immune Defic Syndr 2000;24: 218-226.

15. Korea Centers for Disease Control. Korea notification of HIV/AIDS in 2017 [cited 2019 Aug 12]. Available from: http://www.cdc.go.kr/CDC/cms/content/mobile/23/139823_view.html (Korean).

16. Korea Centers for Disease Control. Korea national policy for HIV/ AIDS management in 2019 [cited 2019 Aug 12]. Available from: http://www.cdc.go.kr/CDC/cms/content/mobile/15/142215_view. html (Korean).

17. Choi BY, Choi JY, Han SH, Kim SI, Kee MK, Kim MJ, et al. Korea HIV/AIDS Cohort Study: study design and baseline characteristics. Epidemiol Health 2018;40:e2018023.

18. Beck AT, Ward CH, Mendelson M, Mock J, Erbaugh J. An inventory for measuring depression. Arch Gen Psychiatry 1961;4:561-571.

19. Spielberger CD, Gorsuch RL, Lushene R, Vagg PR, Jacobs GA. State-trait anxiety inventory for adults: sampler set manual, instrument and scoring guide; 1983 [cited 2019 Oct 16]. Available from: http://www.mhshelps.com/download/State%20Trait%20Anxiety%20Inventory%20Sampler%20Set%20Instrument%20and%20Scoring%20Guide.pdf.

20. Kim JT. Relationship between trait anxiety and sociality [dissertation]. Seoul: Korea University; 1978 (Korean).

21. Mugavero MJ. Improving engagement in HIV care: what can we do? Top HIV Med 2008;16:156-161.

22. CASCADE Collaboration. Survival after introduction of HAART in people with known duration of HIV-1 infection. Lancet 2000; 355:1158-1159.

23. Becker JT, Kingsley LA, Molsberry S, Reynolds S, Aronow A, Levine AJ, et al. Cohort profile: recruitment cohorts in the neuropsychological substudy of the Multicenter AIDS Cohort Study. Int J Epidemiol 2015;44:1506-1516.

24. Kim JM, Choi JY, Jeong WY, Seong H, Kim SW, Kim WJ, et al. Mode of human immunodeficiency virus transmission in Korea: the Korea HIV/AIDS Cohort Study. Korean J Med 2018;93: 379-386 (Korean).

25. Rintamaki LS, Davis TC, Skripkauskas S, Bennett CL, Wolf MS. Social stigma concerns and HIV medication adherence. AIDS Patient Care STDS 2006;20:359-368.

26. Ivanova EL, Hart TA, Wagner AC, Aljassem K, Loutfy MR. Correlates of anxiety in women living with HIV of reproductive age. AIDS Behav 2012;16:2181-2191.

27. Gonzalez A, Solomon SE, Zvolensky MJ, Miller CT. The interaction of mindful-based attention and awareness and disengagement coping with HIV/AIDS-related stigma in regard to concurrent anxiety and depressive symptoms among adults with HIV/AIDS. J Health Psychol 2009;14:403-413.

28. Rueda S, Gibson K, Rourke SB, Bekele T, Gardner S, Cairney J, et al. Mastery moderates the negative effect of stigma on depressive symptoms in people living with HIV. AIDS Behav 2012;16:690-699.

29. Ciesla JA, Roberts JE. Meta-analysis of the relationship between HIV infection and risk for depressive disorders. Am J Psychiatry 2001;158:725-730.

30. Capron DW, Gonzalez A, Parent J, Zvolensky MJ, Schmidt NB. Suicidality and anxiety sensitivity in adults with HIV. AIDS Patient Care STDS 2012;26:298-303.

31. Vyavaharkar M , Moneyham L, Murdaugh C, Tavakoli A. Factors associated with quality of life among rural women with HIV disease. AIDS Behav 2012;16:295-303.

32. Greeff M, Uys LR, Wantland D, Makoae L, Chirwa M, Dlamini P, et al. Perceived HIV stigma and life satisfaction among persons living with HIV infection in five African countries: a longitudinal study. Int J Nurs Stud 2010;47:475-486.

33. Vance DE. Self-rated emotional health in adults with and without HIV. Psychol Rep 2006;98:106-108.

34. Ammassari A, Antinori A, Aloisi MS, Trotta MP, Murri R, Bartoli L, et al. Depressive symptoms, neurocognitive impairment, and adherence to highly active antiretroviral therapy among HIV-infected persons. Psychosomatics 2004;45:394-402.

35. Cherner M, Masliah E, Ellis RJ, Marcotte TD, Moore DJ, Grant I, et al. Neurocognitive dysfunction predicts postmortem findings of HIV encephalitis. Neurology 2002;59:1563-1567.

36. Meade CS, Graff FS, Griffin ML, Weiss RD. HIV risk behavior among patients with co-occurring bipolar and substance use disorders: associations with mania and drug abuse. Drug Alcohol Depend 2008;92:296-300.

37. Nakimuli-Mpungu E, Musisi S, Mpungu SK, Katabira E. Clinical presentation of bipolar mania in HIV-positive patients in Uganda. Psychosomatics 2009;50:325-330.

38. Reid S, Dwyer J. Insomnia in HIV infection: a systematic review of prevalence, correlates, and management. Psychosom Med 2005; 67:260-269.

39. Rubinstein ML, Selwyn PA. High prevalence of insomnia in an outpatient population with HIV infection. J Acquir Immune Defic Syndr Hum Retrovirol 1998;19:260-265.

40. Kim NJ. Trend in CD4+ cell counts at diagnosis in human immunodeficiency virus-infected persons in Korea. Infect Chemother 2017;49:155-157.

41. Centers for Disease Control and Prevention (CDC). Missed opportunities for earlier diagnosis of HIV infection--South Carolina, 1997-2005. MMWR Morb Mortal Wkly Rep 2006;55:1269-1272.

42. Kim MS, Shin SY, Park YS, Kim YA, Ku NS, Kim JH, et al. Therapeutic response of HAART and analysis of related factors in Korean HIV-infected persons. Infect Chemother 2007;39:142-150 (Korean).

Volume: 41, Article ID: e2019037
https://doi.org/10.4178/epih.e2019037

Table 1. General characteristics of research participants

variables	N(%)
Sex	
Men	1,345(93.3)
Women	97(6.7)
Marital status at HIV diagnosis	
Single	770(53.4)
Married / living together	364(25.2)
Separation / divorce / bereavement	125(8.7)
Others	141(9.8)
Missing	42(2.9)
Marital status at baseline	
Single	754(52.3)
Married / living together	353(24.5)
Separation / divorce / bereavement	153(10.6)
Others	140(9.7)
Missing	42(2.9)
Acute symptom of HIV	
Yes	805(55.8)
No	578(40.1)
Unknown	43(3.0)
Missing	16(1.1)
Transmission mode	
Homosexual	493(34.2)
Bisexual	378(26.2)
Heterosexual	491(34.0)
Transfusion / blood products	5(0.3)
Injected drug use	1(0.1)
Vertical transmission	0(0.0)
Others	5(0.3)
Unknown/no answer	70(4.9)
Transmission place	
Korea	889(61.6)
Abroad	161(11.2)
Unknown	392(27.2)

Volume: 41, Article ID: e2019037
https://doi.org/10.4178/epih.e2019037

Table 2. Characteristics of antiretroviral therapy (ART)

	N(%)	Median (IQR)
Antiretroviral therapy		
Yes	1,285(89.1)	
No	157(10.9)	
Initial antiretroviral therapy regimen		
2 NRTI[1] + PI[2]	716(55.7)	
2 NRTI + NNRTI[3]	372(28.9)	
2 NRTI + INI[4]	138(10.7)	
Others [5]	57(4.4)	
Unknown	2(0.2)	
Frequency of skipping antiretroviral drugs (multiple responses)		
Never once	2,225(56.6)	
Once a month	532(13.5)	
Once a week	217(5.5)	
Once in 2 weeks	208(5.3)	
More than twice a week	118(3.0)	
Daily	33(0.8)	
Others	269(6.8)	
Missing	328(8.4)	
Cause of skipping antiretroviral therapy (multiple responses)		
Side effects of antiretroviral drugs	14,318(91.1)	
Fear of revealing diagnosis to others	834(5.3)	
Low socioeconomic status	71(0.4)	
Too many pills	45(0.3)	
Not eating a meal	58(0.3)	
Simply forgot	15(0.1)	
Misinformation about ART	11(0.1)	
Others	9(0.1)	
Missing	359(2.3)	
CD4 T cell count (within 3 months)		
at HIV diagnosis	852(59.0)	229.5(85.5-360.5)
at start of initial ART	563(43.8)	179(60-279)
at finish of initial ART	559(45.0)	331(185-465)
HIV RNA (within 3 months)		
at HIV diagnosis	800(55.4)	63,207.5(14,731.5-224,834.0)
at start of initial ART	524(40.8)	77,100(20,200-240,000)

Volume: 41, Article ID: e2019037
https://doi.org/10.4178/epih.e2019037

at finish of initial ART	539(41.9)	41(20-342)

[1] NRTI (Nucleoside Reverse Transcriptase Inhibitor); [2] PI (Protease Inhibitors);

[3] NNRTI (Non-Nucleoside Reverse Transcriptase Inhibitor); [4] INI (Integrase Inhibitors);

[5] Others; NRTI, 2 NRTI, NRTI + PI, PI, NRTI + NNRTI, 3 NRTI + PI, NRTI + INI + PI, NRTI + NNRTI + PI, 3 NRTI, INI, NNRTI + PI

Table 3. Characteristics of AIDS related disease (include opportunistic infection)

	Total prevalence rate (n=1,442)
**AIDS related/defining diseases [1]**	
Tuberculosis (mycobacterium, non-mycobacterium)	240(16.6)
Syphilis	135(9.4)
Candidiasis, oropharyngeal (thrush)	65(4.5)
Pneumocystis carinii pneumonia (PCP)	53(3.7)
Cytomegalo virus (CMV) disease	39(2.7)
Herpes zoster	28(1.9)
Candidiasis, esophageal	22(1.5)
Herpes simplex	20(1.4)
Herpes simplex virus : bronchitis, pneumonia, or esophagitis	20(1.4)
Peripheral neuropathy	9(0.6)
HIV-related encephalopathy	7(0.5)
Wasting syndrome due to HIV	6(0.4)
Kaposi's sarcoma	5(0.3)
Idiopathic thrombocytopenic purpura	4(0.3)
Burkitt's lymphoma	4(0.3)
Pneumonia (recurrent)	4(0.3)
Candidiasis,vulvovaginal	3(0.2)
Cryptococcus,extrapulmonary	2(0.1)
Cryptosporidiosis, chronic intestinal (>1 month)	2(0.1)
Immunoblastic lymphoma	2(0.1)
Progressive multifocal leukoencephalopathy	1(0.1)
Toxoplasmosis of brain	1(0.1)
Cervical dysplasia (moderate or severe)	1(0.1)
**Opportunistic infection [2]**	
Pneumocystis carinii pneumonia (PCP)	386(26.7)
Candida	155(10.7)
Cytomegalovirus	76(5.3)
Cryptococcus neoformans	8(0.6)
Toxoplasma	4(0.3)
Salmonella	2(0.1)

[1] Diagnosis experience since a year before HIV diagnosis to survey date

[2] A lifelong diagnosis experience regardless of the time of HIV diagnosis

No frequency of occurrence of Bacillary angiomatosis, Listeriosis, Pelvic inflammatory disease, Candidiasis of bronchi, trachea, or lungs, Coccidiodomycosis, disseminated or extrapulmonary, Isosporiasis, chronic intestinal (>1 month's duration), Herpes simplex virus with chronic ulcer(s) (>1 month's duration), Histoplasmosis, disseminated or extrapulmonary, Salmonella septicemia (recurrent), Cervical cancer (invasivea), Primary lymphoma of brain.

Table 4. Characteristics of termination survey

variables	N(%)
Termination rate	509(35.3)
Cause of termination	
Loss to follow up (over 2 years)	152(29.9)
Withdraw consent	95(18.7)
Death	84(16.5)
Withdrawal of the hospital's research	70(13.8)
Changing hospital	52(10.2)
Immigration	1(0.2)
Others	55(10.8)
Cause of death	
AIDS related death	15(17.9)
Non-AIDS related death	7(8.3)
Unknown/missing	62(73.8)

Volume: 41, Article ID: e2019037
https://doi.org/10.4178/epih.e2019037

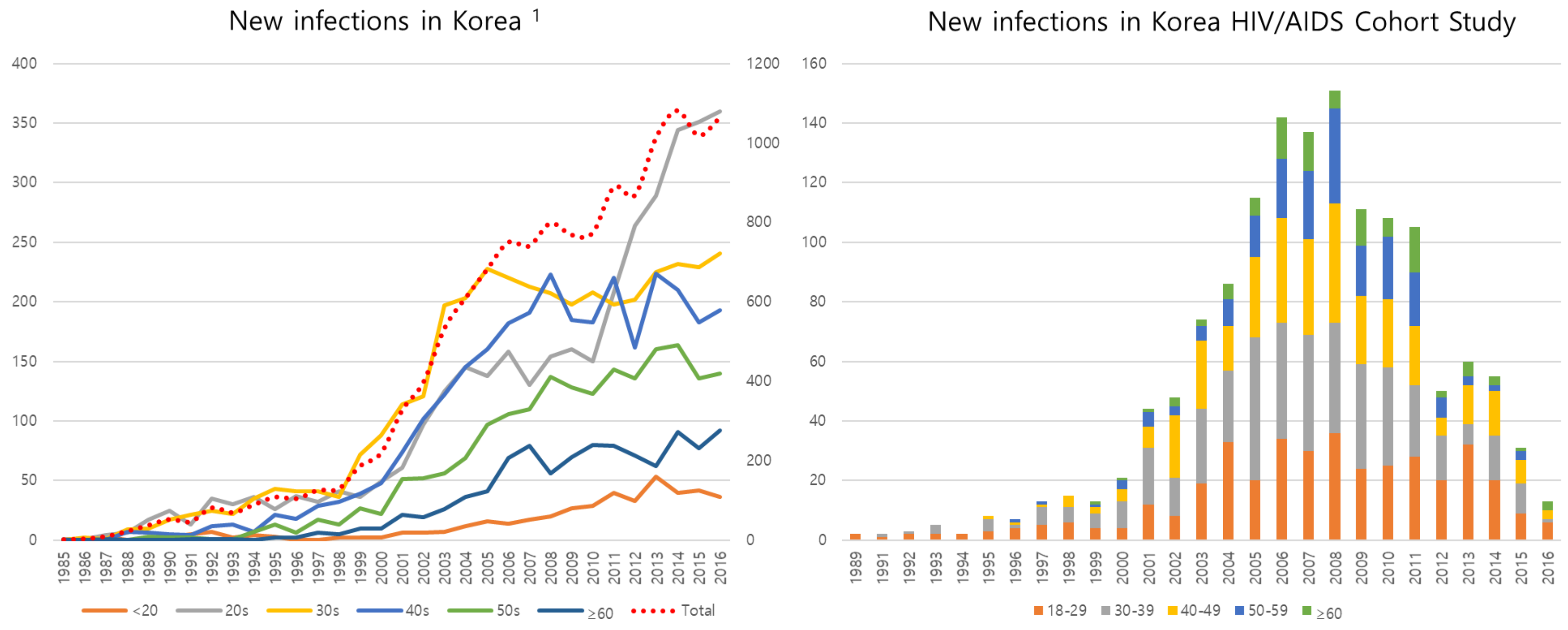

[1] From KCDC, *Notification of HIV/AIDS In Korea*. 2017 [15].

Figure 1. Annual new HIV infections by age

Volume: 41, Article ID: e2019037
https://doi.org/10.4178/epih.e2019037

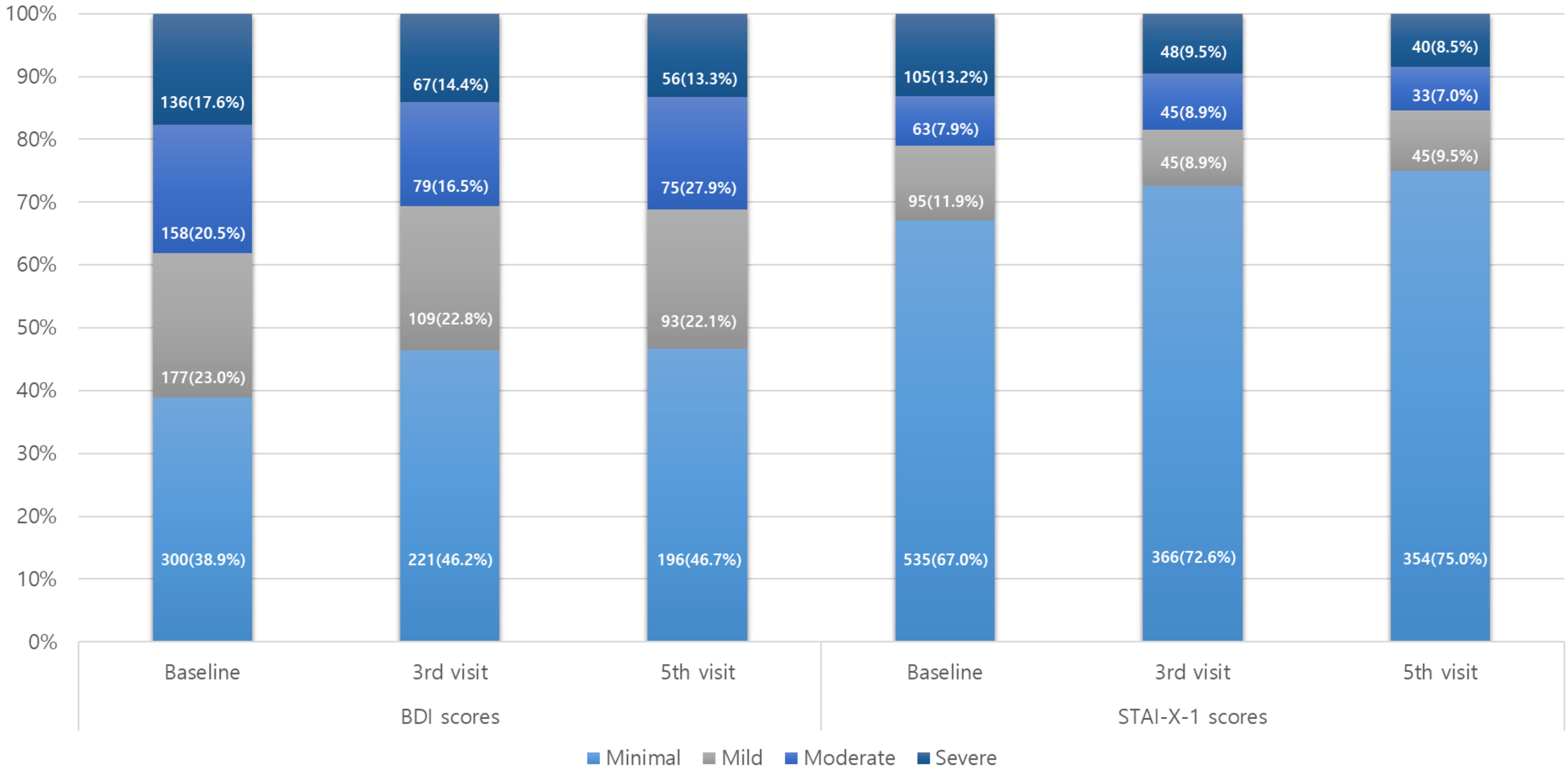

BDI score: Minimal; <10, Mild; 10-15, Moderate; 16-23, Severe; ≥24
STAI-X-1 score: Minimal; <52, Mild; 52-56, Moderate; 57-61, Severe; ≥62

Figure 2. Depressed and anxious status of HIV-infected persons

Volume: 41, Article ID: e2019037
https://doi.org/10.4178/epih.e2019037

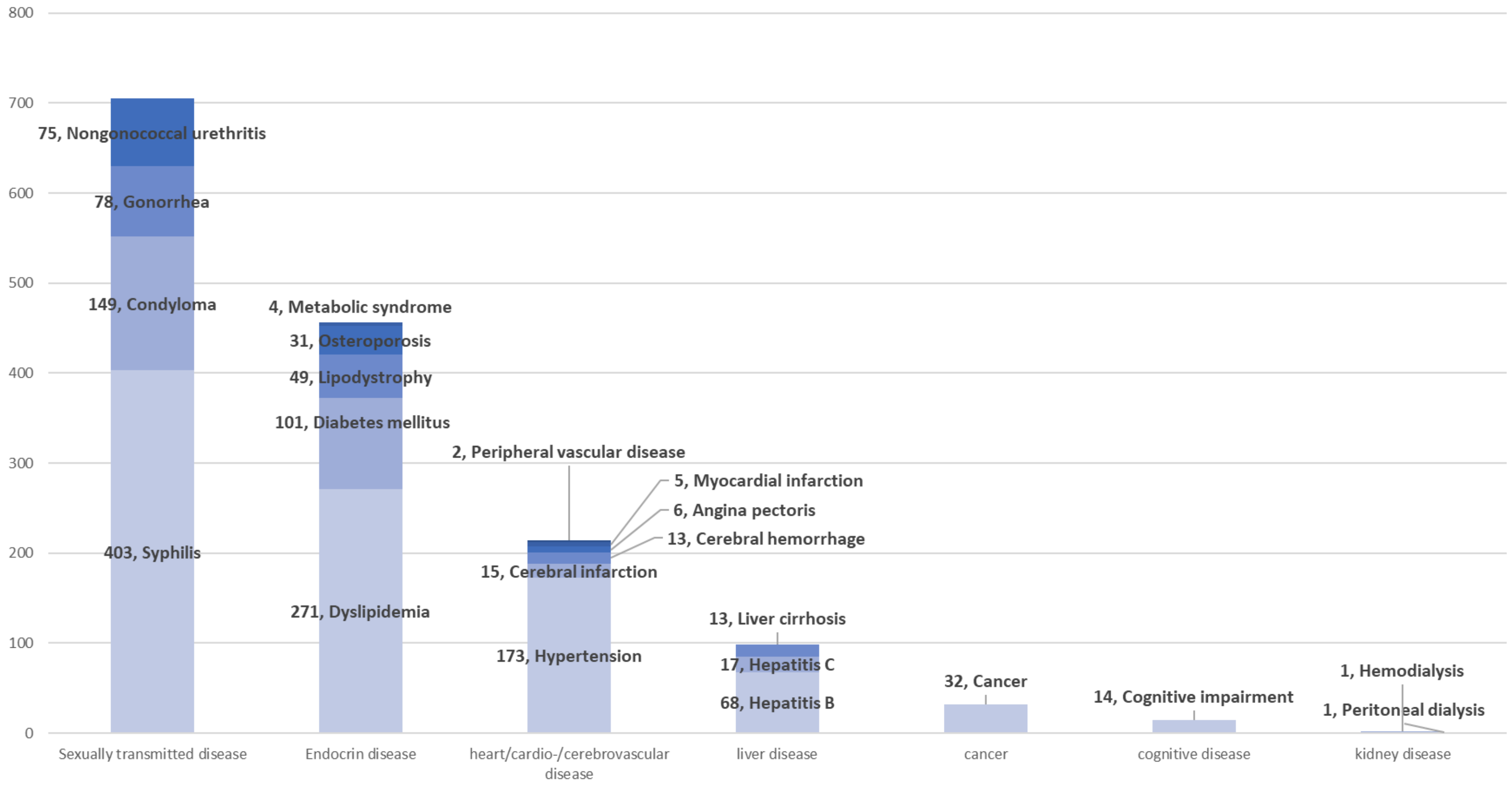

Figure 3. Prevalence of Non-AIDS related diseases
